# Predicting Mortality in Hospitalized COVID-19 Patients in Zambia: An Application of Machine Learning

**DOI:** 10.1155/2023/8921220

**Published:** 2023-05-22

**Authors:** Clyde Mulenga, Patrick Kaonga, Raymond Hamoonga, Mazyanga Lucy Mazaba, Freeman Chabala, Patrick Musonda

**Affiliations:** ^1^Department of Epidemiology and Biostatistics, University of Zambia, Lusaka, Zambia; ^2^Institute of Basic and Biomedical Sciences, Levy Mwanawasa Medical University, Lusaka, Zambia; ^3^The Health Press, Zambia National Public Health Institute, Lusaka, Zambia; ^4^Communication Information and Research, Zambia National Public Health Institute, Lusaka, Zambia

## Abstract

The coronavirus disease 2019 (COVID-19) has wreaked havoc globally, resulting in millions of cases and deaths. The objective of this study was to predict mortality in hospitalized COVID-19 patients in Zambia using machine learning (ML) methods based on factors that have been shown to be predictive of mortality and thereby improve pandemic preparedness. This research employed seven powerful ML models that included decision tree (DT), random forest (RF), support vector machines (SVM), logistic regression (LR), Naïve Bayes (NB), gradient boosting (GB), and XGBoost (XGB). These classifiers were trained on 1,433 hospitalized COVID-19 patients from various health facilities in Zambia. The performances achieved by these models were checked using accuracy, recall, *F*1-Score, area under the receiver operating characteristic curve (ROC_AUC), area under the precision-recall curve (PRC_AUC), and other metrics. The best-performing model was the XGB which had an accuracy of 92.3%, recall of 94.2%, *F*1-Score of 92.4%, and ROC_AUC of 97.5%. The pairwise Mann–Whitney U-test analysis showed that the second-best model (GB) and the third-best model (RF) did not perform significantly worse than the best model (XGB) and had the following: GB had an accuracy of 91.7%, recall of 94.2%, *F*1-Score of 91.9%, and ROC_AUC of 97.1%. RF had an accuracy of 90.8%, recall of 93.6%, *F*1-Score of 91.0%, and ROC_AUC of 96.8%. Other models showed similar results for the same metrics checked. The study successfully derived and validated the selected ML models and predicted mortality effectively with reasonably high performance in the stated metrics. The feature importance analysis found that knowledge of underlying health conditions about patients' hospital length of stay (LOS), white blood cell count, age, and other factors can help healthcare providers offer lifesaving services on time, improve pandemic preparedness, and decongest health facilities in Zambia and other countries with similar settings.

## 1. Introduction

Infectious diseases have always shaped the world in many ways, from changing the rules that govern daily life to restricting movement and travel and thereby disrupting daily life to the point of bringing the entire world to a total standstill. This has been very evident in the COVID-19 pandemic, which has claimed millions of lives since its outbreak [1]. This study [2] focuses on COVID-19 mortality in Zambia and how predicting mortality can improve public health preparedness and save lives.

COVID-19 is caused by the Severe Acute Respiratory Syndrome Coronavirus 2 (SARS-CoV-2). The COVID-19 pandemic has challenged how the field of public health handles typical infectious diseases and how it conducts research. At the time of this writing and according to the data reported by the Johns Hopkins University Center for Systems Science and Engineering [3], the pandemic had already affected the global population with some 610, 200, 000 cases and 6, 500, 000 deaths; in Africa, about 12, 060, 000 cases and 256, 000 deaths; and in Zambia, with over 333,  000 cases and 4,  000 deaths [4]. The situation became extremely overwhelming and attracted the attention of researchers from various fields of the research community.

Zambia has experienced surging COVID-19 cases and mortalities on a national scale. This has heavily overwhelmed local communities and especially public health facilities which have proven to be ill-prepared since the start of the pandemic. One of the major challenges Zambia faced was pandemic unpreparedness which has been shown to be an essential factor in the fight to control any pandemic [5]. Failure to predict COVID-19 mortality in patients with the greatest risk posed a public health challenge towards unpreparedness which in turn caused improper prioritization, underestimation, and underallocation of funds towards the government's pandemic response plan [6].

Many research studies have been done on COVID-19 pandemic so far using both traditional statistical methods and ML techniques [7]. There have been a few past studies that used ML algorithms for COVID-19 mortality classification. A study that compared two prediction models based on statistical and computational ML algorithms to predict mortality in COVID-19 hospitalized patients [8] found that between a conventional statistical method of LR and a ML method of artificial neural network (ANN) when validated on 16 features against a sample of 482 laboratory-confirmed COVID-19 hospitalized patients, the ANN achieved the best performance with an ROC_AUC of 90%. However, despite the high performance, the authors of the study acknowledged the limitations associated with having used only two ML algorithms, having conducted the study at a single center and on merely 482 participants, which affected the generalizability of their findings. The authors also acknowledged that there were no efforts to address the misclassification bias that may have been potentially introduced by the class imbalance that existed between 382 (79.25%) who recovered and 100 (20.74%) who succumbed to the disease, in which case the use of Synthetic Minority Oversampling Technique (SMOTE) should have been performed. Another study conducted on 1710 hospitalized COVID-19 patients developed and evaluated several ANNs to predict the mortality risk in hospitalized COVID-19 patients. The backpropagation artificial neural network (BP-ANN) was the best model and achieved an ROC_AUC of 88.8%. For this study, the authors acknowledged the limitations presented by the single-center nature of their selected dataset, and the use of only two ANN algorithms in different configurations.

Although this research was not focused on proposing totally new methods and procedures, there are a few components that represent the novelty of our study in addressing certain gaps identified in past studies. In order to improve the generalizability of our findings, we aimed to target a much larger study sample that included participants from multiple healthcare facilities. Most studies have not implemented several ML methods simultaneously and have thus recommended the use of several ML methods in order to have a clear picture of how these algorithms perform when compared with each other. In addition, the procedure for picking the best model in most of the studies reviewed simply pick the ML model with the highest value in the metric being considered, and there are no follow-up attempts to determine if the difference observed visually between two competing algorithms is actually statistically significant. To address this concern, this study sought to develop and validate several ML algorithms that included the following: (1) ecision tree (DT), (2) random forest (RF), (3) support vector machines (SVM), (4) logistic regression (LR), (5) Naïve Bayes (NB), (6) gradient boosting (GB), and (7) XGBoost (XGB). These algorithms were implemented simultaneously after which the procedure for selecting the best model used pairwise comparisons of each model compared to all other models for the various metrics used as explained in the post hoc analysis section of the Materials and Methods section. This helped in determining whether the differences observed visually between two competing models were actually statistically significant. This also made it possible for this study to have a statistical basis for proposing and recommending multiple ML algorithms as alternatives to the top performing model in situations where there were no statistically significant differences observed between the best model and the second-best model, something that is hardly done in ML research.

This study was conducted in order to help Zambia's healthcare system prepare for current and future pandemics and sought to predict mortality in hospitalized COVID-19 patients using ML. It employed a special form of ML called supervised ML [9]. The use of ML in this study was chosen due to a number of reasons. Progress in computer science and technology has made the application of ML in public health research to become more common today. As it has been observed, ML models have been preferred in situations involving extremely dynamic datasets, automation, and greater computing abilities [10, 11]. This study thus sought to develop an ML pipeline that supports automation, reusability, and reproducibility. ML algorithms have also been shown in a number of studies [12] to possess improved and unmatched performance as these models continually improve significantly as more data become available over time. Another advantage that favored the use of ML in this research was that, while most conventional statistical methods are proficient at detecting simpler univariate and multivariate associations, it often requires more sophisticated ML algorithms to detect complex interactions and heterogeneous feature associations in which different unspecified subgroups of instances in the data may have distinct underlying feature associations with outcome [13].

This research is intended to answer two fundamental questions:

- Based on Zambia's COVID-19 data for the period March 2020 to October 2021, how accurately can mortality be classified among hospitalized COVID-19 patients?
- What fundamental factors among those collected by Zambia's health facilities hugely influence COVID-19 patients' susceptibility to mortality?

The main objective of this study was to derive and validate supervised ML models to predict mortality in hospitalized COVID-19 patients in Zambia. This main objective was further subdivided into three (3) specific objectives:

- Perform internal validation for mortality prediction on the COVID-19 dataset for the period of March 2020 to October 2021 in Zambia
- Quantify the influence that features have on the final mortality outcome among hospitalized COVID-19 patients in Zambia from March 2020 to October 2021
- Check the performance evaluation metrics for each of the candidate models used in predicting mortality to assess the level of success achieved by each model

This paper is organized into six main sections. The Introduction section contains the background of the study and the description of the research problem and objectives. The Literature Review has highlighted important studies that successfully used similar methods in addressing COVID-19 mortality. The Materials and Methods section has provided important guidelines about the design of the study and the various ML methods implemented in the analysis. The Results section presents the results of the various tests conducted. These results are then discussed in detail in the Discussion section. The Conclusion section has outlined the conclusions drawn from the study and the recommendations proposed for further research. Extra supplementary materials are also provided and are listed and described in the Supplementary Materials section.

## 2. Literature Review

This section presents the review of literature published in various journals on COVID-19 mortality. The literature considered were searched from the MEDLINE database using the PubMed online search engine. For each research paper reviewed, the focus was on the study design and setting, study sample, study purpose, methods, and main results.

The first part of the literature review presents papers that have addressed factors that contribute to severe COVID-19 and mortality [11, 14, 15]. The second focuses on studies that attempted to predict mortality in COVID-19 patients using ML methods and the associated performances for various evaluation metrics. The final part of our literature review has addressed a few studies that have compared ML models with conventional statistical models in order to appreciate why ML models were chosen for this study.

First and foremost, there have been a number of studies that have described predictors of severe COVID-19, which could probably be in the causal pathway leading to mortality. In a study entitled: *Risk factors for mortality in critically ill patients with COVID‐19 in Huanggang, China: A single‐center multivariate pattern analysis* [16], a group of researchers outlined multiple risk factors that led to severe COVID-19 and even death in a number of extreme cases. The paper observed 192 critically ill COVID-19 patients in which 142 survived and 50 died in hospital. After data were compared between survivors and nonsurvivors, and performing multivariate pattern analysis to identify possible risk factors for COVID-19 mortality, several factors were identified. These included age, duration (time from illness onset to admission), Barthel index score, whether laboratory examination indicators included C-reactive protein, white blood cell count, platelet count, fibrin degradation products, oxygenation index, lymphocyte count, and D-dimer. In another study (*COVID-19 mortality risk assessment: An international multicenter study*), Bertsimas et al. [17] addressed many more risk factors of severe COVID-19 and mortality including age, sex, heart rate, heart disease, diabetes, chronic kidney disease, cardiac dysrhythmias, and a few other features. These features were derived from a population of 3,062 COVID-19 patients. The mortality rate was 26.84%. In comparison to survivors, nonsurvivors were older with a median age of 80, whereas survivors had a median age of 64. Of the nonsurvivors, men were 67.2% while women were only 58.4%. It was also reported that the prevalence of comorbidities such as cardiac dysrhythmias, chronic kidney disease, and diabetes were higher in the nonsurvivor population versus the survivor population (9.61%, 4.21% and 15.92% versus 5.56%, 1.74%, and 11.42%, respectively). In all these studies with varying study settings and study samples, a few features have appeared in many multiple studies. These are age, sex, hospitalization, pneumonia, acute respiratory distress syndrome, HIV, TB, malignancy, diabetes, cardiac disease, hypertension, chronic pulmonary disease (CPD), chronic kidney disease (CKD), and chronic lung disease. These features were thus targeted in this study.

After the review of the literature that attempted to predict mortality in COVID-19 patients, the following studies were reviewed. Josephus et al. [18] conducted a study on 114 Indonesian COVID-19 patients, and the objective of the study was to make mortality predictions on COVID-19 patients with nonmedical features. The study used a single LR model which achieved an accuracy of more than 90%; further analysis found that age was the most important predictor of patient's mortality. The author recommended a larger study sample as only 114 patients were used. It was also noted that more ML methods were missing with which comparisons should have been made in order to choose the best model. In a different study conducted in China involving a cohort of 2,160 participants analysed retrospectively, Gao et al. [19] built an ensemble mortality risk prediction model for COVID-19 using four ML methods including LR, SVM, Gradient-Boosted-DT, and ANN. The results found that the ensemble model achieved an ROC_AUC of 0.9621 (95% CI: 0.9464–0.9778). Some of the limitations acknowledged by the authors included the fact that participants were primarily only local residents from Wuhan, China, and recommended investigation of the predictive performance of the ML models in other regions and ethnicities and the evaluation of the prognostic implications of the ensemble ML model in prospective cohorts other than the retrospective cohorts used in their study. In another retrospective study in South Korea involving 3,524 patients, Das et al. [20] conducted a study to predict mortality among confirmed COVID-19 patients in South Korea using machine learning. Of the five ML algorithms (LR, SVM, KNN, RF, and GB) used, the results showed that LR was the best model and achieved an ROC_AUC of 83.0%. There were a number of limitations reported by the authors including unavailability of crucial clinical information on symptoms and risk factors. A major setback reported by the author of this study was the reuse of a subset of data for validation that was also included in the cross-validation process. This may have led to overfitting of the models with the available data. We also noticed that despite the extreme class imbalance in their dataset which contained 3,529 (97.9%) cases and 74 (2.1%) deaths, there were no efforts to address the potential misclassification bias that may have been introduced by this imbalance, in which case balancing of the outcome classes using SMOTE should have been inevitable. In a much bigger multinational cross-sectional study involving a huge sample of 2,670,000 participants from 146 countries, Pourhomayoun and Shakibi [21] designed and developed several ML models (SVM, ANN, RF, DT, LR, and K-Nearest Neighbor (KNN)) to determine the health risk of patients with COVID-19. The study results found the best model to be the RF which achieved an ROC_AUC of 94.0%. This was a high-quality study with huge study samples; however, the performance was not exceptional; this could have been due to various confounding variables and other complex feature interactions that may have crept into the study due to the huge differences in population characteristics across national or regional boarders; thus, results may have been stratified according to regions having countries with similar population characteristics.

Some studies have compared ML models with conventional statistical models in prediction problems, in which ML models were preferred to conventional statistical models. One study entitled: *Comparison of Conventional Statistical Methods with Machine Learning in Medicine: Diagnosis, Drug Development, and Treatment* [22]. The study was a narrative review whose aim was to offer an expert perspective on the comparison of traditional statistical methods with ML, and their corresponding advantages and limitations in medicine, with a specific focus on the integration between the two approaches and its application to illness detection, drug development, and treatment. It compared the usefulness and limitations of traditional statistical methods and ML, when applied to the medical field. This study recommended a method that best meets the requirements and best solves the problem at hand. It also recommended a hybrid approach that integrates both ML and traditional approaches if doing so can add beneficial results to the study.

The current review of the literature suggests that the use of ML in medical research has not been fully utilized despite the advantages associated with its use. Moreover, the newness of ML models and their heavy reliance on programming skills have added to the complicatedness of ML models and hindered most researchers from using ML methods where they ought to be used. This has resulted in less applicability of ML models. Reproducibility and consistency have always been the anchors of evidence-based medical research; however, the way in which most ML research studies have been documented has made it harder to reproduce ML methods. Thus, this study aimed to address a number of issues identified in the various studies reviewed. These issues involved the use of larger study samples from various locations to improve generalizability of findings, the implementation of several ML methods from which the best model should be chosen, the use of SMOTE in inevitable situations having extreme class imbalance of outcome categories to remove misclassification bias, and the use of a statistical procedure for selecting the best model that performs significantly better than other models. The ML methods used in this study were intended to improve the reusability of ML pipelines built in order to allow others to apply similar methods to similar classification problems.

## 3. Materials and Methods

This section discusses the various methods used in the study, which are the design and setting of the study, data analysis, and the type of ethical approval obtained.

**Figure 1:**
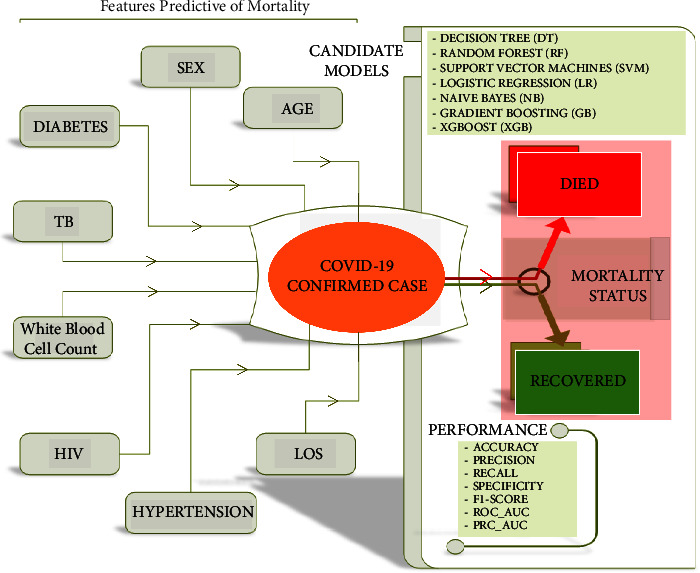
Visual research conceptual framework.

This study followed the standard guidelines of a typical ML research outlined by Luo et al. [23] in the paper *“Guidelines for Developing and Reporting Machine Learning Predictive Models in Biomedical Research: A Multidisciplinary View.”* A visual conceptual framework displayed in Figure 1 was developed to visually display the outcome (COVID-19 mortality) and various features that are predictive of mortality.

**Figure 2:**
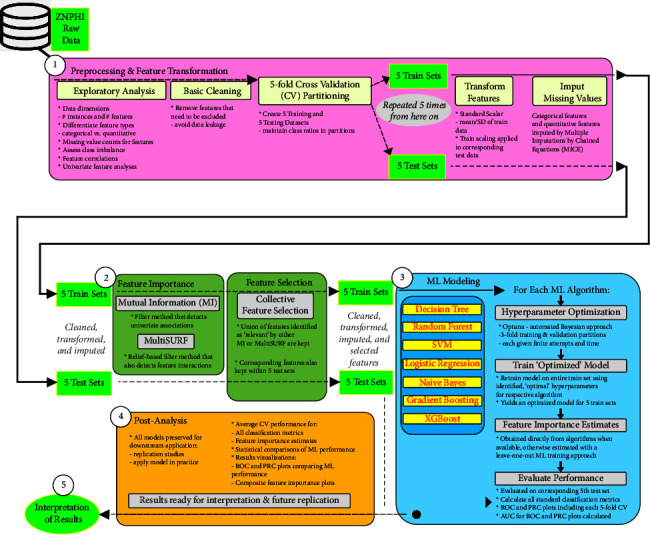
Machine learning modelling steps.

To further expand the research conceptual framework, a more detailed visual graphic of the machine learning modelling steps implemented in this research was adapted from Urbanowicz et al. [13] and is shown in Figure 2.

### 3.1. Study Design

The outcome of interest and the exposures in this study were analysed simultaneously, and study participants were selected only based on relevance to the study objectives and not on the status of the outcome nor exposures. This qualified the study to use an analytical cross-sectional design as recommended by Wang and Cheng [24].

### 3.2. Study Population and Study Setting

This study was conducted in Zambia which is estimated to have a population of about 18 million with the majority of the people (98%) estimated to be under the age of 65 years of age [25, 26]. This is an important observation since age is an essential predictor of COVID-19 mortality. The study population targeted included all confirmed cases of COVID-19 that were hospitalized in various health facilities in Zambia from the period of March 2020 to October 2021. The data used were from the Zambia National Public Health Institute (ZNPHI), which house the combined datasets from various health institutions that were selected by the Ministry of Health to be COVID-19 centres across the country.

### 3.3. Measurement Variables

The measurement variables used in this study were chosen based on recent studies [16, 17] that have showed that COVID-19 in the presence of a number of comorbidities is more likely to lead to mortality. Thus, the comorbidities chosen included age, diabetes, tuberculosis, and other underlying conditions, as listed in Table 1.

### 3.4. Eligibility Criteria

This research targeted the data collected by ZNPHI from various health facilities in Zambia for which all confirmed COVID-19 cases hospitalized during the period of March 2020 to October 2021 were eligible to be included in the study. However, pregnant women were excluded from the study due to the variable vaccination status for which there was no acceptable vaccine for pregnant women in Zambia at the time this research was conducted. Other excluded cases involved records that had too many missing variables for which the application of multiple imputations would have simply added extra noise to the dataset.

### 3.5. Handling of Missing Data

Since the study applied ML models that do not allow missing values in the dataset, missing values needed to be imputed for the models to run. The Supplementary Material of “Figure S2: Dataset missingness map” contains details on the level of missingness associated with each of the features used. Multiple imputations by chained equations (MICE) [27] was the method used to impute missing values using the *mice* package in R [28].

### 3.6. Handling of Bias

**Figure 3:**
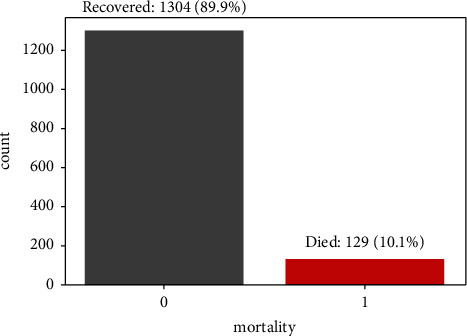
Proportion of patients who recovered and patients who died.

It was also noted that there was a large class imbalance in the proportions of the patients who recovered and the proportion of patients who died, as shown in Figure 3. Extreme class imbalance has been widely reported by many ML experts to have the potential to introduce misclassification bias or type II error [29, 30]. This prompted the use of Synthetic Minority Oversampling Technique (SMOTE) [31, 32] to balance the label classes in the dataset. Results from an imbalanced dataset were then compared to the results from a SMOTE-balanced dataset in order to check if balancing classes really helped the ML classifiers in reducing the type II error.

### 3.7. Data Analysis

This section described the various software packages used in this study, the classification models used, and the performance evaluation metrics employed. The data analysis used all the data that were made available by ZNPHI, involving 1,433 COVID-19 hospitalized patients.

#### 3.7.1. Statistical Software Packages Used

The Python programming language version 3.8.0 [33] and its libraries scikit-learn version 1.1.0 [34] and XG Boost [35] were used in ML model development. The integrated development environment (IDE) used included JupyterLab version 3.4.0 [36] and Visual Studio Code version 1.70.0 [37]. Other minor exploratory analyses were conducted using R version 4.2.0 [38], the recent easy-to-use statistical software packages jamovi version 2.3.16 [39], and JASP version 0.16.2.0 [40].

#### 3.7.2. Validation Strategy

Due to feasibility and resources constraints associated with external validation of ML models on a new independent dataset [41], this study only performed internal validation for the developed ML models. The dataset was split into the training and test sets in the ratio 80 : 20 using 5-fold cross-validation strategy, which has been shown to be sufficient in assessing the generalization ability of ML models [42].

The ML models used were optimized for performance using the various scikit-learn and XG Boost hyperparameter tunings [43, 44]. The ML models were all trained and tested on the same dataset, after which the performance evaluation metrics were assessed to identify the best-performing model. Before running the candidate models, a Pearson correlation analysis of all pairs of features was conducted to identify potentially redundant or highly correlated features, followed by a univariate correlation analysis between outcome and individual features where numerical features were analysed using the chi-square test of independence and categorical features were analysed using the Mann–Whitney U test; this helped in consolidating the feature importance analysis that followed after.

#### 3.7.3. The Decision Tree (DT) Algorithm

The DT model [45] is a type of supervised ML algorithm used in classification problems in which the model follows a set of if-else conditions to either visualise the data or classify it in accordance with the possible outcomes presented. This study implemented the categorical variable DT during the mortality classification process. The model used the decision tree classifier from the scikit-learn library with hyperparameter tunings shown in the Supplementary Material for “ML Models Optimization Hyperparameter” in Table S1 [46].

#### 3.7.4. The Random Forest (RF) Algorithm

The RF algorithm [47, 48] is an ensemble learning method that combines many DTs and averages them to make a final decision. This produces a more complex and powerful classifier. The RF model uses the random forest classifier from the scikit-learn library and is implemented with hyperparameter attributes shown in the Supplementary Material for “ML Models Optimization Hyperparameter” in Table S2 [49].

#### 3.7.5. The Support Vector Machine (SVM) Algorithm

The SVM [50] is a classification algorithm in which each data point is plotted in the n-dimensional space by using support vectors, which are the coordinates corresponding to each individual data point, where *n* is the number of features that best differentiates the two classification classes. The SVM algorithm performs classification by using the SVC (support vector classifier) from the scikit-learn library. The SVC separates the data into their classes using the right hyperplane using the hyperparameters shown in the Supplementary Material for “ML Models Optimization Hyperparameter” in Table S3 [51].

#### 3.7.6. The Logistic Regression (LR) Algorithm

The LR model can be defined as the ML algorithm that is applied in classification problems using the concept of probability in predictive analysis by assigning observations a logistic cost function termed as a sigmoid function *σ*(*z*)=(1/1+*e*^−*z*^) that maps predicted values to their associated probabilities ranging from 0 to 1; it penalises the model for every wrong prediction and works towards reducing those misclassification errors [52]. The LR model is a linear model that uses the logistic regression classifier from the scikit-learn library with hyperparameter attributes presented in the Supplementary Material for “ML Models Optimization Hyperparameter” in Table S4 [53].

#### 3.7.7. The Naïve Bayes (NB) Algorithm

The NB model [54] is a classification method that uses the popular Bayesian method of prior likelihood in the implementation of classification. It is based on Bayes theorem, which states that if an outcome event is partitioned into *k* nonintersecting (mutually exclusive or independent) categories *B*_1_,  *B*_2_,…, *B*_*k*_, then the probability of an *i*^*th*^ event *B*_*i*_ happening given an event *A* is given by the following equation:

(1) $$\begin{array}{c} P\left(B_{i}|A\right)=\frac{P\left(A|B_{i}\right).P\left(B_{i}\right)}{P\left(A|B_{1}\right).P\left(B_{1}\right)+P\left(A|B_{2}\right).P\left(B_{2}\right)+\cdots +P\left(A|B_{k}\right).P\left(B_{k}\right)}. \end{array}$$

Classification by the NB algorithm was implemented using the Gaussian NB classifier (Gaussian Naive Bayes) from the scikit-learn library, making the assumption that the likelihoods of features are assumed to be Gaussian such that parameters *σ*_*y*_ and *μ*_*y*_ are estimated using the method of maximum likelihood. Since the NB classifier is naturally less complex, all hyperparameters of the Gaussian NB classifier were left to be run with their default attributes [55].

#### 3.7.8. The Gradient Boosting (GB) Algorithm

The GB model [56] builds an additive model in a forward stagewise fashion; it allows for the optimization of arbitrary differentiable loss functions. In each stage, *n_classes* regression trees are fit to the negative gradient of the binomial or multinomial deviance loss function. Binary classification is a special case where only a single regression tree is induced. The optimization of the gradient boosting classifier was achieved by hyperparameter tuning shown in the Supplementary Material for “ML Models Optimization Hyperparameter” in Table S5 [57].

#### 3.7.9. The XGBoost (XGB) Algorithm

The extreme gradient boosting algorithm, popularly known as XGBoost [35], is an ensemble ML model that employs the gradient boosting framework during classification tasks and provides parallel tree boosting. This study implemented the XGB using the XGB classifier with the optimization hyperparameter tunings shown in the Supplementary Material for “ML Models Optimization Hyperparameter” in Table S6 [44].

#### 3.7.10. Performance Evaluation Metrics

The metrics used to evaluate the performance of models in this study were accuracy, recall (sensitivity), and specificity. In order to get a clearer picture of the models' performance that is free from bias from the imbalance between classes in the dataset, the analysis of the areas under the ROC and PRC curves were prioritized. To supplement the use of accuracy, the *F*1-score was used to optimize the trade-off between precision and recall [58].

### 3.8. Post Hoc Analysis

At the end of achieving the desired results, a procedure for determining the best model was proposed to go beyond simply picking the ML model with the highest value in the metric being considered. The best model was determined by first conducting nonparametric statistical analyses to compare the averages of the performance evaluation metrics for every pair of the ML models used. Secondly, an analysis was done to determine which of the ML models had evaluation metrics that yielded significant findings in the Kruskal–Wallis one-way analysis of variance (*p* value ≤0.05). Finally, those models were then run through follow-up pairwise Mann–Whitney U-tests to compare between all possible pairs of the seven ML models used to identify the existence of a significant difference in performance. Thus, for each metric assessed, the number of all possible pairwise ML model combinations from the seven algorithms used resulted in 21 combinations (computed from *C*(7, 2)=(7!)/((7 − 2)!×2!)=21). The best model was then picked based on the existence of a statistically significant difference between a number of competing models. If multiple outstanding models are competing and the pairwise Mann–Whitney U-tests do not show the existence of a statistically significant difference, then choosing the model whose metric has the highest value as the best model should be accompanied by the argument that, in the event that the top model could not be implemented, the other competing models should be used with the same confidence as though they were the best model.

### 3.9. Ethical Approval

This study was approved by the University of Zambia Biomedical Research Ethics Committee (UNZABREC), approval number: REF-2106-2021. This study was also registered with the National Health Research Authority (NHRA) of Zambia, reference number: NHRA-00009-06-01-2022. All essential requirements as requested for in Zambia were met and commitment to uphold all ethical guidelines regarding confidentiality and proper handling of the patient's confidential medical records was ensured.

## 4. Results

This section presents the various findings of this study. The summary statistics presented describe the characteristics of the data used, followed by the results of the feature importance analysis and the results of the classification models presented with their performance evaluation metrics.

### 4.1. Sample Characteristics

The sample involved 1, 433 hospitalized COVID-19 patients with a variety of characteristics. The overall mean age and standard deviation of the entire sample was 50.5 (16.3). The study sample included 911 (63.6%) males and 512 (36.4%) females. The proportion of COVID-19 admitted patients who died from the disease was 129 (10.1%), while the proportion of those who recovered from the disease formed a majority class of 1304 (89.9%) patients.

**Figure 4:**
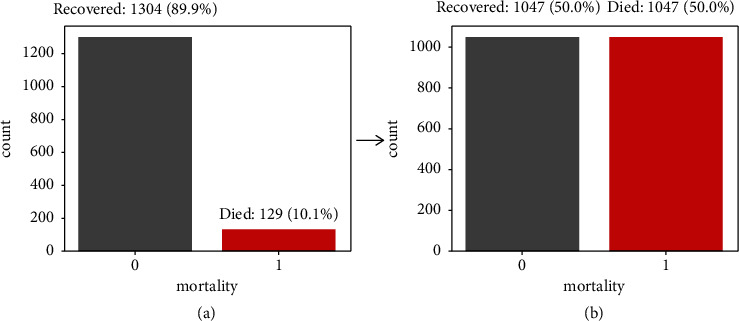
Proportions of two mortality classes. (a) Imbalanced mortality classes. (b) Classes balanced by SMOTE.

The results of performing SMOTE on the imbalanced dataset produced a balanced dataset shown in Figure 4.

Table 2 presents a summary of numerical features in the form of averages and medians with their respective standard deviations (SD) and interquartile ranges (IQR) where appropriate.  Table 3 presents a summary of categorical features with their respective proportions in percentages.

The results in Table 2 describe the numerical features of the study participants which all showed a strong significant association with COVID-19 mortality. The median number of days spent in the hospital (LOS) for patients who recovered and those who died was 4.0 (IQR = 5.0) days and 2.0 (IQR = 2.0) days, respectively. For the feature age as it was expected, the mean age of those who died was as high as 56.6 (SD = 17.1) years, whereas 49.9 (SD = 16.1) years was the mean age of those who survived. It can also be seen that the median white blood cell count for patients who died was 7.8 (IQR = 8.5) cells per *μ*L, whereas patients who recovered recorded 6.7 (IQR = 4.6) cells per *μ*L. The results in Table 3 also describe eleven categorical features of the study participants. Five of these features (diabetes, hypertension, wave, ward, and CPD) showed a strong significant association with COVID-19 mortality, whereas the other features did not show a significant association with COVID-19 mortality.

### 4.2. Feature Importance Analysis

**Figure 5:**
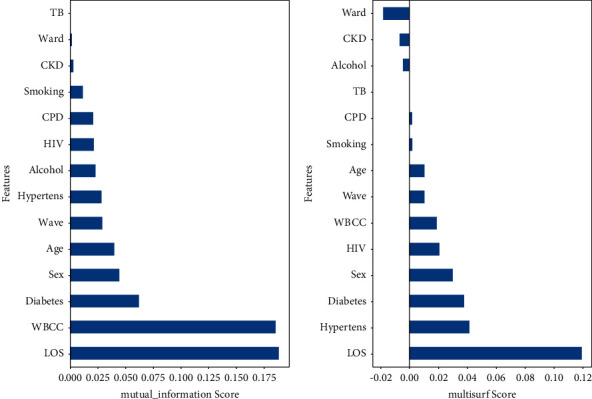
Feature importance analysis. (a) Mutual information scores. (b) Multisurf scores.

The results of a feature importance analysis in Figure 5 show both the mutual information scores and the multisurf scores. The mutual information score highly ranked LOS and white blood cell count with an approximate score of 0.188. Other relatively important features in order of decreasing importance included diabetes, sex, age, wave, and hypertension. The multisurf scores, on the other hand, showed which of the important features were given maximum priority, and what features were given the least priority. The first priority was primarily given to LOS with a relatively high score of 0.12, whereas the second priority was given to the features hypertension, diabetes, sex, HIV, white blood cell count, wave, and age (in descending order of importance). On the other hand, chronic kidney disease (CKD), alcohol intake, tuberculosis, and admission ward were not prioritized.

**Figure 6:**
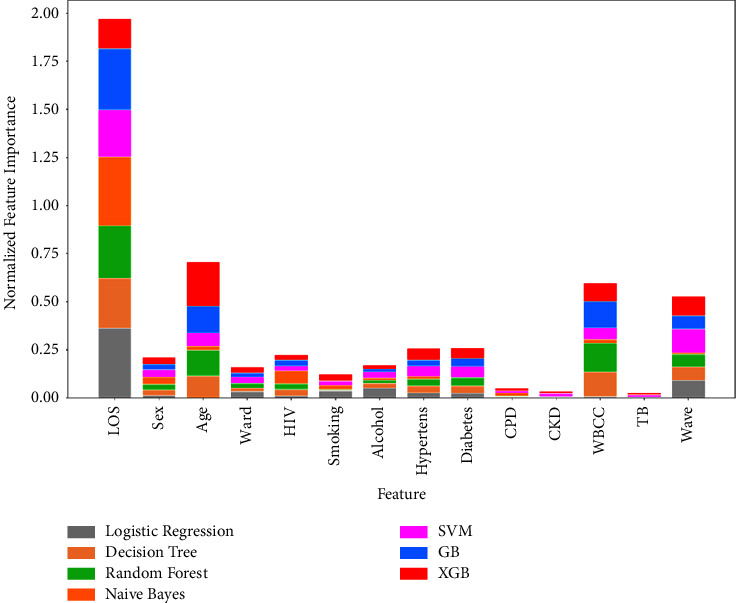
Normalized compound feature importance plot.

Figure 6 presents the normalized compound feature importance plot in the form of stacked bar graphs. The size of the portion of the bar for each ML model represents the proportional contribution of each ML model in comparison to the total magnitude of importance that each feature was given. In harmony with the mutual information scores and the multisurf scores, the normalized compound feature importance plot for the seven algorithms used also confirmed that LOS stood out as the most influential feature with a score of almost 2.00. This was followed by an approximate score of 0.70 for age, white blood cell count, and wave.

The results of the feature importance analysis complemented the results of the univariate feature analysis and guided the removal of some features that had little influence on the classification of mortality.

### 4.3. Performance of Classification Models

The results of the seven ML models used in this study are now presented and include both the results from imbalanced and balanced mortality classes. The results have also presented the performance of models that used all features compared to those that used only selected important features.

**Figure 7:**
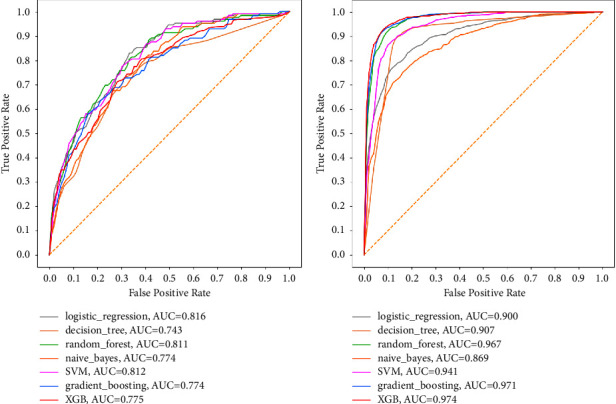
ROC_AUC of models for selected features. (a) For imbalanced classes. (b) For balanced classes.

To begin with, the results of ML models using the ROC_AUC are displayed in Figure 7. It was observed that for the dataset with imbalanced classes, ML models performed relatively well with ROC_AUC values ranging from 0.743 to 0.816, where LR was the best model and DT was the underperforming model. However, it was observed that despite maintaining the same hyperparameter tunings, ROC_AUC results improved significantly for all seven models when mortality classes were balanced using SMOTE, with ROC_AUC values now ranging from as high as 0.869 to a whopping 0.974, where the XGB was the best model whereas the NB was the underperforming model.

**Figure 8:**
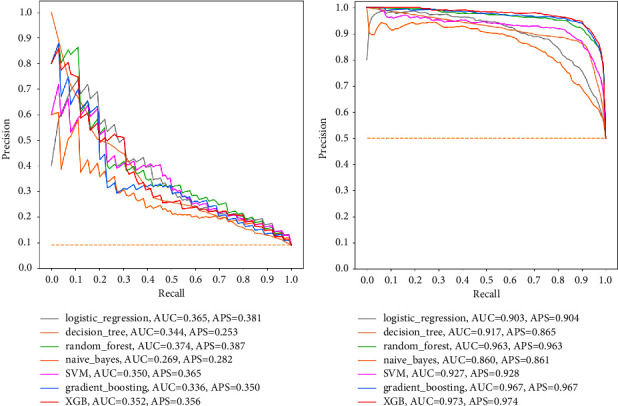
PRC_AUC for selected features. (a) Imbalanced classes. (b) Balanced classes.

Secondly, the results of ML models using the PRC_AUC are now presented in Figure 8. It was observed that for this relatively unbiased metric, all seven models performed unacceptably poor and worse for the dataset with imbalanced classes. The PRC_AUC results ranged poorly from 0.269 to 0.365, where RF was the best model, whereas NB was the underperforming model. In a surprising turn of events, despite maintaining the same hyperparameters tunings for all models, PRC_AUC results showed tremendous performance improvements for the dataset where mortality classes were balanced using SMOTE. PRC_AUC results now ranged from 0.860 to 0.973. The best model in PRC_AUC results for the balanced dataset was now the XGB while the underperforming model was the NB.

Thirdly, having compared the performance improvements of the seven models as indicated by the ROC and PRC plots, it was clear that balancing mortality classes using SMOTE led to better performance improvements for all models used. Following the use of the dataset with balanced classes as a better choice for removing bias, the study then sought to determine whether all fourteen features assumed to be predictive of COVID-19 mortality were helping the models perform better. This led to the removal of some features that were less important and less predictive of mortality, as was earlier shown by the mutual information , multisurf , and normalized feature importance scores. This resulted in a series of trials that led to the removal of five less influential features: smoking, alcohol, chronic pulmonary disease (CPD), chronic kidney disease (CKD), and TB.

**Figure 9:**
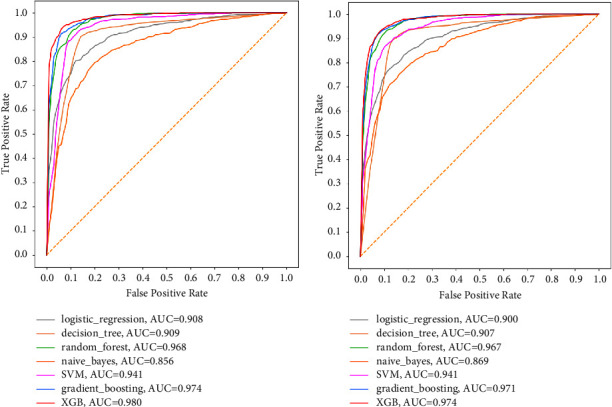
ROC_AUC for balanced classes. (a) All features. (b) Selected features.

The results of models with all fourteen features compared to models with only selected features using ROC_AUC as the evaluation metric are now presented in Figure 9. Models that used selected features only left out five less influential features (smoking, alcohol, CPD, CKD, and TB).

It can be clearly seen from Figure 8 that there are no significant differences in the performance of the seven models when the ROC_AUC results for all features are compared with the ROC_AUC results for the selected features. This invoked the use of the law of parsimony, which favours the model with fewer features.

Finally, performance results of ML classifiers were now evaluated using various metrics including accuracy, recall (sensitivity), specificity, precision, ROC_AUC, and PRC_AUC, as presented in Table 4. The performance results of the seven ML models used are presented in descending order starting from the best-performing model to the worst-performing model: XGB, GB, RF, SVM, DT, LR, and NB.

The post hoc analysis of performance metric results for each ML model yielded significant results from the Kruskal–Wallis one-way analysis of variance. This result validated the analysis of a follow-up pairwise Mann–Whitney U-test for each metric. In order to determine the best model from the seven ML models used, this study concentrated on comparing the ROC_AUCs and checking whether a significant difference existed between each pair since similar results were also observed in other evaluation metrics checked.

The pairwise Mann–Whitney U-test analysis comparing ROC_AUC results showed that despite the average algorithm performance in ROC_AUC being 93.3%, the algorithms NB, LR, and DT performed significantly worse ( *p* value ≤0.05) than the other ML models used. It was also found that the SVM algorithm performed significantly better than NB, LR, and DT; however, it still performed significantly worse than the top three models (RF, GB, and XGB). As presented in Table 4, among the top three performing models, the best model was the XGB with ROC_AUC of 98.2% for all features and 97.5% for selected features; it was followed by the GB, which had ROC_AUC of 97.6% for all features and 97.1% for selected features; it was also followed by the RF in third place with ROC_AUC of 96.9% for all features and 96.8% for selected features. Further observation found that the pairwise Mann–Whitney U-test analysis of the top three models did not show any significant difference between the best-performing model (XGB) and the second-performing model (GB); there was also no significant difference between the XGB as the best model and the RF as the third-performing model.

## 5. Discussion

This section now discusses the results just presented and offers appropriate interpretations of the findings. A brief summary of the findings is presented first, followed by a discussion of important features that hugely influenced patients' susceptibility to mortality. Finally, the discussion of the performance evaluation metrics for the ML models to guarantee the quality of the predictions made is presented.

### 5.1. Summary of Findings

This study aimed to apply supervised ML models to predict mortality in hospitalized COVID-19 patients in Zambia by deriving and validating seven (7) ML models for mortality prediction on Zambia's COVID-19 dataset. The study successfully performed internal validation on the dataset and identified features that proved to be predictive of mortality. It was found that hospital length of stay and blood cell count can effectively help in determining mortality; knowledge of patients' ages and diabetes status was also found to be reasonably useful. The study then quantified the influence that predictive features have on the final mortality outcome among hospitalized COVID-19 patients. The findings showed that the features used can be ranked in order of decreasing importance, starting with hospital length of stay as the most influential feature, followed by age, wave, diabetes, hypertension, and sex, respectively. The performance of the ML models used was then checked to identify the model that fitted the data best. The findings showed that the XGB model outperformed all other models in the performance evaluation metrics used having an ROC_AUC of 97.5%, followed by the GB model, which performed significantly lower than the best model and had an ROC_AUC of 97.1%, whereas the worst-performing model (NB) equally had a reasonably good ROC_AUC of 86.9%. This meant that the XGB model fitted the dataset better than other models and was thus recommended in this study.

### 5.2. Feature Importance

The feature importance analysis used three effective methods: the mutual information score, the multisurf score, and the normalized compound feature importance plot. The results of these analyses noted that all three methods consistently and unanimously gave coherent findings about the features that were most important and predictive of COVID-19 mortality. The most important feature that was found to be the most predictive of mortality was hospital length of stay, followed by white blood cell count. It was clearly seen that these two features were very important and greatly influenced how the ML models classified the mortality status of a COVID-19 patient. Other influential factors arranged in order of decreasing importance included age, wave, diabetes, hypertension, and sex.

The implications of the feature importance analysis findings show that if healthcare providers know exactly the factors adding to the length of hospitalization of a patient and if they have full knowledge of a hospitalized patient's age and sex and the type of variant (represented by the variable wave) and whether the patient is diabetic or hypertensive, then they can well estimate the possibility of a COVID-19 case deteriorating into a severe disease or mortality. This knowledge can also help government agencies responsible for public health to secure enough funding that can be used in implementing measures that prioritise the healthcare of hospitalized COVID-19 patients that have the highest risk of mortality in Zambia. This can also be applied in other countries with a similar setting as Zambia.

### 5.3. ML Model Performance

This discussion is focused on the results of ML models that were run on selected features since the conditions for which a parsimonious model should be preferred were satisfied. Firstly, it was found that the application of SMOTE to balance the classes in the dataset was extremely essential and significantly improved the performance of the ML models across all performance evaluation metrics used. This was evidently observed in the metric precision (PPV) for which most of the ML models fared poorly. For the dataset with imbalanced mortality classes, the two worst-performing models were DT, which had the precision of 19.1% and 19.8% for all features and selected features, respectively, and SVM, which had the precision of 19.1% and 20.2% for all features and selected features, respectively. However, after the mortality classes in the dataset were balanced using SMOTE, the performance of the ML classifiers improved significantly such that the DT recorded a precision of 87.4% and 86.4% for all features and selected features, respectively, whereas the SVM recorded a precision of 87.4% and 85.6% for all features and selected features, respectively. This study thus recommends the use of SMOTE in ML classification problems in which class imbalances are huge enough to introduce potential misclassification bias.

All the ML models used in this study achieved reasonably high performance as compared to other studies presented in the Literature Review section. As presented in Table 4, the top three ML models that achieved outstanding performance for the balanced dataset using selected features were the XGB, GB, and RF. The other ML models, such as the SVM, DT, LR, and NB, also achieved similar results despite those results being significantly lower when compared to the top three models as observed from the pairwise Mann–Whitney U-test analysis.

The results of the post hoc analysis helped to establish that the best-performing model in this study, the XGB classifier, together with the second-best model, the GB, and the third-best model, the RF, did not differ significantly, since it was shown that both the GB and the RF did not perform significantly worse than the XGB. This implies that the top three models of our study, the XGB, GB, and RF are all best suited for the dataset used and can thus be recommended in similar classification problems in which higher performance is sought to be achieved.

The implications of the reasonably high performances recorded by the ML algorithms used can greatly help in future modelling of COVID-19 data. Since all seven ML models used performed reasonably well, future modelling of COVID-19 mortality may have to seriously consider the models used with special attention given to the XGB model as the most effective in mortality predictions for hospitalized COVID-19 patients. Other models that may have to be considered are the GB and the SVM models. The application of these ML models may have serious implications for effectively and accurately predicting COVID-19 mortality including other similar health conditions which may greatly help in the control of both current and future pandemics.

### 5.4. Comparison of Findings with Other Studies

The findings of this study were consistent with other studies, like those presented in the literature review. Current literature indicates that factors such as age, diabetes, hypertension, sex, and HIV are predictive of COVID-19 mortality. This was clearly evident in the findings of this study where LOS, age, white blood cell count, and type of variant (wave) were shown to be influential in helping classify the mortality status of the participants. Furthermore, like other studies have shown, ML models can be very powerful in modelling how factors associated with COVID-19 mortality can help in the classification of the health outcome in hospitalized patients. The performance of ML models for various evaluation metrics under proper conditions and with the right hyperparameter tunings can achieve higher values for accuracy, precision, ROC_AUC, PRC_AUC, and other metrics as clearly observed in this study, although it is not unusual to record poor results for some models if the data do not fit such models well.

### 5.5. Interesting Findings

This study also yielded some interesting findings discussed in this section. It has not commonly been seen in most studies that the LOS of admitted patients is an important feature in most classification problems of COVID-19 mortality. This could be due to the fact that the variable LOS is rarely collected since it varies for every day a patient remains admitted to a health facility. Surprisingly, LOS was the most important variable in the dataset used, and this was observed for all seven algorithms validated. Another feature which was ranked as the second most important was the white blood cell count. This also came as a surprise, as it has not been frequently used in most of the classification models as the literature review indicated. The reason for the rare use of the variable white blood cell count seems to also be associated with the rare events in which the variable is collected.

The feature “wave” was deliberately chosen to represent the type of COVID-19 variant that is on the rampage and was equally shown to be predictive of COVID-19 mortality. The feature “ward” was also predictive of mortality. On the other hand, the features smoking, alcohol, chronic pulmonary disease (CPD), chronic kidney disease (CKD), and TB were not shown to be important, and removing them did not significantly affect the performance of the ML models.

### 5.6. External Validity of Findings

The methods implemented in this study and the results found may be effectively applied to various study settings other than the Zambian setting in which this study was conducted.

The participants selected for this study, as described in the eligibility criteria, involved every hospitalized confirmed COVID-19 case with an exception of pregnant women only. Thus, participants included various individual traits that were characteristic of the various health facilities in Zambia from which they came. This led to a reasonably large study sample that was highly inclusive, representative, and free from potential sources of sampling bias, which in turn added to the external validity of the study. The generalization ability of the ML models used was also strengthened by the use of the 5-fold cross-validation strategy as recommended by Berrar [42]. This study also followed strictly the strong ML methodologies, standards, and guidelines proposed by Luo et al. [23], making it possible for any researcher to easily apply our methods to reproduce our findings in another study setting similar to the one in which this study was conducted by reusing our ML pipeline codes available on the open science framework through the links provided in the supplementary materials section.

### 5.7. Strengths and Limitations of Study

As seen from the higher performances obtained from the ML models used, this can be attributed to the quality of the methods used and how they conform to the standards of ML guidelines, methodological procedures, and conventions. This section discusses some of the strengths and limitations associated with our study.

This study used proven methodological procedures and well-documented guidelines, such as those recommended by Urbanowicz et al. [13], for the various hyperparameters proposed after a number of trials and simulations. The level of automation associated with the ML pipeline that was created for this study has enabled our ML algorithms to be almost completely reproducible in similar settings upon the availability of a dataset. This may greatly help similar studies that may need to reproduce the results presented or employ similar methods in another study setting. Since the study sample was large and participants came from various health facilities of Zambia, this has made the findings of our study to be more generalizable as compared to other studies. Despite the huge class imbalance observed in the dataset, the use of SMOTE significantly reduced misclassification bias in the study and led to increased performance of ML models. Another strength of our study was our use of multiple ML models and the use of a statistical procedure in selecting the best-performing model.

It is now important to also weigh the limitations associated with our study. There were two major limitations in our study. The first limitation was due to having a higher percentage of missingness (18%), as shown in the Supplementary Material of “Figure S1: dataset missingness map.” Despite the use of the MICE procedure to handle missing values, it has been shown that imputing a dataset that has a higher percentage of missingness may introduce noise into the dataset. Thus, similar studies would record performance improvements if a dataset with a lower percentage of missingness was used. The second limitation was that most of the Zambian health facilities lack effective screening and diagnostic test equipment, which hinders the collection of well-known clinical features that have been shown to be predictive of COVID-19 mortality. Similar studies that seek to reproduce our findings should involve several clinical features that were missing in our study to improve the quality and reliability of the results.

## 6. Conclusion

Predicting mortality in hospitalized COVID-19 patients using factors that have an influence on the severity of the health condition is an essential undertaking in public health and epidemiology. In conclusion, it can be reasonably stated that, like other studies have shown, the classification models of XGB, GB, RF, SVM, DT, LR, and NB successfully achieved the primary objective of this study by effectively showing their strength in predicting mortality in 1,433 hospitalized patients in Zambia using the features collected from patients with reasonably higher values of accuracy, recall (sensitivity), specificity, precision, F1 Score, ROC_AUC, and PRC_AUC. The findings obtained, if put to use, have the potential to improve preparedness in health facilities, proper prioritization of funds, and healthcare to save the lives of COVID-19 patients with the greatest risk of mortality.

Having successfully derived and validated the seven ML models that achieved sufficiently higher performances, it can be concluded that the XGB classifier, which was chosen to be the ideal and best-performing model, performed well in our classification problem and that it should be highly considered in classification problems in similar settings. It can also be added that the GB and RF can also be effective alternatives to the XGB for similar studies. It has been seen that there are many factors that were shown to influence the susceptibility of hospitalized COVID-19 patients to mortality. The factors LOS and white blood cell count strongly influenced the classification process, while other factors like age, sex, hypertension, diabetes, and ward also showed noticeable influence in determining the mortality outcome. This implies that healthcare providers should be fully aware of underlying health conditions of their patients in order to offer lifesaving services that may help in both improving preparedness and decongesting health facilities.

### 6.1. Recommendations for Public Health Practice and Further Research

Having stressed the importance of factors that are predictive of COVID-19 mortality, we greatly recommend that health facilities where COVID-19 patients are admitted should carefully and accurately keep track of each patient's LOS and also collect patients' white blood cell count, in addition to other routine variables discussed in this study. There should be sustained prioritization of admitted patients that are identified as having the greatest risk of mortality, and vaccination should be encouraged as soon as it is necessary. Due to the drawbacks associated with the interpretability of ML models [59], this study also recommends that similar studies try to use a hybrid approach that uses both ML and conventional statistical classification methods to help in having more interpretable results that will go beyond identifying features as important but also describe the nature of the influence on the classification problem, that is, whether the predictive features identified increased or reduced mortality and with what value they either increased or reduced mortality. This would powerfully combine the advantages associated with both methods regarding high performance and having interpretable findings.

To add to the body of knowledge and consolidate the findings obtained in this study, especially the interesting findings stated, we greatly recommend studies that might simply aim to reproduce the findings of this study in another study setting. The success of such studies would help to firmly accept the interesting findings of this study as reproducible and reliable.

## Figures and Tables

**Table 1:** Study variables.

Type of variable	Variables	Scale of measurement
Outcome variable	Mortality	Binary

Predictor variables	Age	Discrete
	LOS	Discrete
	White blood cell count	Continuous
	Sex	Categorical
	Admission ward	Categorical
	HIV status	Categorical
	Tuberculosis	Categorical
	Smoking	Categorical
	Alcohol	Categorical
	Diabetes	Categorical
	Hypertension	Categorical
	Chronic pulmonary disease	Categorical
	Chronic kidney disease	Categorical

**Table 2:** Summary of numerical features.

Numerical features	Total (1,433)	Recovered (1,304)	Died (129)	*P* value
LOS (days)	4.9 (4.2)^a^	4.0 (5.0)^b^	2.0 (2.0)^b^	<0.001^c^
Age (years)	50.5 (16.3)^a^	49.9 (16.1)^a^	56.6 (17.1)^a^	<0.001^c^
White blood cell count (/*μ*L)	6.720 (4.9)^b^	6.7 (4.6)^b^	7.8 (8.5)^b^	<0.001^c^

Note.^a^Mean (SD), ^b^median (IQR); ^c^*p* value from chi-square test.

**Table 3:** Summary of categorical features with their respective proportions.

Categorical features	Subcategories	Total: 1,433 (100%) *n* (%)	Recovered: 1,304 (89.9%) *n* (%)	Died: 129 (10.1%) *n* (%)	*P* value
Sex	Male	911 (63.6)	828 (63.5)	83 (64.3)	0.925^d^
	Female	522 (36.4)	476 (36.5)	46 (35.7)	

HIV	Positive	276 (19.3)	251 (19.2)	25 (19.4)	1.000^d^
	Negative	1157 (80.7)	1053 (80.8)	104 (80.6)	

Diabetes	Yes	352 (24.6)	303 (23.2)	49 (38.0)	<0.001^d^
	No	1081 (75.4)	1001 (76.8)	80 (62.0)	

Hypertension	Yes	729 (50.9)	644 (49.4)	85 (65.9)	<0.001^d^
	No	704 (49.1)	660 (50.6)	44 (34.1)	

Wave	1^st^-wave	534 (37.3)	506 (38.8)	28 (21.7)	<0.001^d^
	2^nd^-wave	566 (39.5)	505 (38.7)	61 (47.3)	
	3^rd^-wave	333 (23.2)	293 (22.5)	40 (31.0)	

Ward	General	496 (34.6)	453 (34.7)	43 (33.3)	<0.001^d^
	Suspect	250 (17.4)	242 (18.6	8 (6.2)	
	Infectious dis.	251 (17.5)	216 (16.6	35 (27.1)	
	High cost	293 (20.4)	277 (21.2	16 (12.4)	
	ICU	143 (10.0)	116 (8.9)	27 (20.9)	

^*∗*^Smoking	Yes	65 (4.5)	64 (4.9)	1 (0.8)	0.054^d^
	No	1368 (95.5)	1240 (95.1)	128 (99.2)	

^*∗*^TB	Yes	68 (4.7)	64 (4.9)	4 (3.1)	0.481^d^
	No	1365 (95.3)	1240 (95.1)	125 (96.9)	

^*∗*^CKD	Yes	32 (2.2)	28 (2.1)	4 (3.1)	0.699^d^
	No	1401 (97.8)	1276 (97.9)	125 (96.9)	

^*∗*^Alcohol	Yes	204 (14.2)	185 (14.2)	19 (14.7)	0.971^d^
	No	1229 (85.8)	1119 (85.8)	110 (85.3)	

^*∗*^CPD	Yes	21 (1.5)	13 (1.0)	8 (6.2)	<0.001^d^
	No	1412 (98.5)	1291 (99.0)	1296 (93.8)	

Note: ^d^*p* value from Mann–Whitney U test; ^*∗*^features omitted in models with selected features.

**Table 4:** Performance of ML models for all features compared to selected features in both imbalance and balanced mortality classes.

		Imbalanced classes		Balanced classes	
		All features	Selected features	All features	Selected features
XGB	Accuracy	0.734	0.718	0.934	0.923
	Recall (TPR)	0.675	0.706	0.945	0.942
	Specificity (TNR)	0.740	0.731	0.923	0.904
	Precision (PPV)	0.218	0.226	0.925	0.907
	*F*1-score	0.325	0.335	0.934	0.924
	ROC_AUC	0.793	0.776	0.982	0.975
	PRC_AUC	0.347	0.353	0.983	0.974

GB	Accuracy	0.623	0.906	0.921	0.917
	Recall (TPR)	0.279	0.279	0.933	0.942
	Specificity (TNR)	0.968	0.968	0.908	0.892
	Precision (PPV)	0.472	0.446	0.911	0.897
	*F*1-score	0.348	0.340	0.922	0.919
	ROC_AUC	0.731	0.774	0.976	0.971
	PRC_AUC	0.289	0.337	0.974	0.967

RF	Accuracy	0.721	0.789	0.907	0.908
	Recall (TPR)	0.636	0.628	0.926	0.936
	Specificity (TNR)	0.807	0.805	0.889	0.880
	Precision (PPV)	0.246	0.248	0.893	0.886
	*F*1-score	0.354	0.352	0.909	0.910
	ROC_AUC	0.811	0.811	0.969	0.968
	PRC_AUC	0.392	0.374	0.966	0.964

SVM	Accuracy	0.705	0.697	0.897	0.880
	Recall (TPR)	0.713	0.784	0.928	0.914
	Specificity (TNR)	0.696	0.689	0.865	0.845
	Precision (PPV)	0.191	0.202	0.874	0.856
	*F*1-score	0.301	0.320	0.900	0.884
	ROC_AUC	0.792	0.813	0.941	0.941
	PRC_AUC	0.340	0.351	0.922	0.928

DT	Accuracy	0.703	0.726	0.880	0.882
	Recall (TPR)	0.713	0.660	0.888	0.906
	Specificity (TNR)	0.702	0.733	0.872	0.857
	Precision (PPV)	0.191	0.198	0.874	0.864
	*F*1-score	0.301	0.303	0.881	0.885
	ROC_AUC	0.752	0.743	0.909	0.907
	PRC_AUC	0.351	0.345	0.922	0.918

LR	Accuracy	0.723	0.718	0.831	0.819
	Recall (TPR)	0.737	0.753	0.840	0.832
	Specificity (TNR)	0.723	0.715	0.823	0.806
	Precision (PPV)	0.210	0.208	0.826	0.812
	*F*1-score	0.326	0.325	0.832	0.821
	ROC_AUC	0.810	0.817	0.908	0.901
	PRC_AUC	0.370	0.365	0.911	0.903

NB	Accuracy	0.886	0.895	0.785	0.792
	Recall (TPR)	0.247	0.225	0.728	0.806
	Specificity (TNR)	0.950	0.962	0.841	0.778
	Precision (PPV)	0.327	0.371	0.821	0.784
	*F*1-score	0.280	0.278	0.770	0.794
	ROC_AUC	0.762	0.774	0.856	0.869
	PRC_AUC	0.266	0.270	0.844	0.860

## Data Availability

Data are not publicly available; however, it may be made available if the data request is approved by ZNPHI.

## Supplementary Materials

Supplementary Materials Supplementary Material 1: supplementary material 1 (PDF file (.pdf), 46.6 kB-“Figure S1-Dataset missingness map.pdf”) is a figure (Figure S1), and it shows the level of missingness shown by a missingness map, which was produced before multiple imputations by chained equations (MICE) were performed. Supplementary material 2: supplementary material 2 (PDF file (.pdf), 176.0 kB-“ML Models Optimization Hyperparameters.pdf”) contains guidelines in table form about how the hyperparameter tunings and their attributes were set in model development to optimize for best performance for each of the seven ML models validated. Table S1 shows the hyperparameters for the decision tree classifier for the DT model. Table S2 contains hyperparameters for the random forest classifier for the RF model. Table S3 contains hyperparameters for the SVC (support vector classifier) for the SVM model. Table S4 contains hyperparameters for the logistic regression classifier for the LR model. Table S5 contains hyperparameters for the gradient boosting classifier for the GB model. Finally, Table S6 contains hyperparameters for the XGB classifier for the XGB model.
