# Supplementary material for: Predicting Mortality in Hospitalized COVID-19 Patients in Zambia: An Application of Machine Learning
Source: Glob Health Epidemiol Genom. 2023 May 22;2023:8921220. doi: 10.1155/2023/8921220 (PMC10228226; doi:10.1155/2023/8921220)
Supplement: Supplementary Materials — Supplementary Material 1: supplementary material 1 (PDF file (.pdf), 46.6 kB-“Figure S1-Dataset missingness map.pdf”) is a figure (Figure S1), and it shows the level of missingness shown by a missingness map, which was produced before multiple imputations by chained equations (MICE) were performed. Supplementary material 2: supplementary material 2 (PDF file (.pdf), 176.0 kB-“ML Models Optimization Hyperparameters.pdf”) contains guidelines in table form about how the hyperparameter tunings and their attributes were set in model development to optimize for best performance for each of the seven ML models validated. Table S1 shows the hyperparameters for the decision tree classifier for the DT model. Table S2 contains hyperparameters for the random forest classifier for the RF model. Table S3 contains hyperparameters for the SVC (support vector classifier) for the SVM model. Table S4 contains hyperparameters for the logistic regression classifier for the LR model. Table S5 contains hyperparameters for the gradient boosting classifier for the GB model. Finally, Table S6 contains hyperparameters for the XGB classifier for the XGB model. [file 8921220.f1.zip › ML Models' Optimization Hyperparameters.pdf]

ML Models Optimization Hyperparameters:

Table S1: DecisionTreeClassifier for DT and its optimization hyperparameters.

#	Hyperparameter attributes	Description
1	'criterion': ['gini', 'entropy']	Measures the quality of a split in the tree. Where *'gini'* is for the Gini impurity and *'entropy'* is for the information gain.
2	'splitter': ['best', 'random']	Strategy used to choose the split at each node. The two options chosen for the DT model are *'best'* to choose the best split and *'random'* to choose the best random split.
3	'max_depth': [1, 30]	Defines the maximum depth of the tree.
4	'min_samples_split': [2, 50]	Describes the minimum number of samples required to split an internal node.
5	'min_samples_leaf': [1, 50]	Reviews the minimum number of samples required to be at each leaf node. A split point at any depth is only considered if it leaves at least the defined *min_samples_leaf* training samples in each of the left and right branches.
6	'max_features': [None, 'auto','log2']	Maximum number of features to consider when looking for the best split. It first of all considers '*None*' for $max\_features = n\_features$, followed by '*auto*' for $max\_features = sqrt(n\_features)$, and finally considers '*log2*' for $max\_features = log2(n\_features)$.
7	'class_weight': [None, 'balanced']	Helps in computing the weights associated with the classes. For *None*, all classes are supposed to have a weight of 1. The '*balanced*' mode uses the values of the outcome to automatically adjust weights inversely proportional to the class frequencies in the input data.

Table S2: RandomForestClassifier for RF and its optimization hyperparameters.

#	Hyperparameter attributes	Description
1	'criterion': ['gini', 'entropy']	Measures the quality of a split in the tree. Where *'gini'* is for the Gini impurity and *'entropy'* is for the information gain.
2	'splitter': ['best', 'random']	Strategy used to choose the split at each node. The two options chosen for the DT model are *'best'* to choose the best split and *'random'* to choose the best random split.

3	'max_depth': [1, 30]	Defines the maximum depth of the tree.
4	'min_samples_split': [2, 50]	Describes the minimum number of samples required to split an internal node.
5	'min_samples_leaf': [1, 50]	Reviews the minimum number of samples required to be at each leaf node. A split point at any depth is only considered if it leaves at least the defined *min_samples_leaf* training samples in each of the left and right branches.
6	'max_features': [None, 'auto','log2']	Maximum number of features to consider when looking for the best split. It first of all considers '*None'* for $max\_features = n\_features$, followed by '*auto'* for $max\_features = sqrt(n\_features)$, and finally considers '*log2'* for $max\_features = log2(n\_features)$.
7	'bootstrap': [True]	Allows the algorithm to use the bootstrap resampling method while building (growing) trees, as opposed to using the entire dataset in each tree built.
8	'oob_score': [False, True]	A conditional hyperparameter that only holds if and only if the bootstrap has been enabled. It permits the algorithm to use out-of-bag samples to estimate the generalization score.
9	'class_weight': [None, 'balanced']	Maintains the same attributes as the DT built earlier that is being extended.

Table S3: SVC (support vector classifier) for SVM and its optimization hyperparameters.

#	Hyperparameter attributes	Description
1	'kernel': ['linear','poly','rbf']	Specifies type of kernel to be used in the algorithm. The three options chosen include: *'linear', 'poly',* and *'rbf'*. From which the hyperparameter optimization sweep will choose the best option that will yield the best performing model.
2	'C': [0.1, 1000]	A regularization parameter whose strength is inversely proportional to C. This value was kept strictly positive and corresponds to the penalty of the square of 12.
3	'gamma': ['scale']	Specifies the kernel coefficient to be used for 'rbf', 'poly' in their use, such that the '*scale'* is default passed in order to use the value $1/((n\_features * X.var()))$.
4	'degree': [1,6]	Defines the degree of the polynomial kernel function '*poly'*.

5	'probability': [True]	Enables use of probability estimates.
6	'class_weight': [None, 'balanced']	Set to two possible options, ‘*None’* for assigning all other classes to the weight of one, or ‘*balanced’* mode to enable outcome values to automatically adjust weights inversely proportional to the class frequencies in the input data.

Table S4: LogisticRegression classifier for LR and its optimization hyperparameters.

#	Hyperparameter attributes	Description
1	'penalty': ['l2','l1']	Specifies the norm used in the penalization. Two options L1 and L2 have been set and the hyperparameter sweep will choose the option that yields the best results.
2	'C': [1e-5, 1e5]	Defines the inverse of regularization strength, the two options set give the model ability to try both a smaller value $1e-5$ for stronger regularization and a larger as an alternative.
3	'solver': ['newton-cg','lbfgs', 'liblinear', 'sag','saga']	Determines which algorithm is chosen in the optimization problem. The hyperparameter sweep attempts multiple options; ‘*liblinear’* for small datasets, ‘*sag’* and *‘saga’* for large datasets since they are faster, including *‘newton-cg’* and *‘lbfgs’,* for handling the penalty of L2.
4	'class_weight': [None, 'balanced']	Set to two possible options, ‘*None’* for assigning all other classes to the weight of one, or ‘*balanced’* mode to enable outcome values to automatically adjust weights inversely proportional to the class frequencies in the input data.
5	'max_iter': [10, 1000]	Sets the maximum number of iterations taken for the solver to converge.

Table S5: GradientBoostingClassifier for GB and its optimization hyperparameters.

#	Hyperparameter attributes	Description
1	'loss': ['deviance', 'exponential']	Describes the loss function to be optimized. ‘*deviance’* refers to deviance (= LR) for classification with probabilistic outputs. For loss ‘*exponential’* GB recovers the AdaBoost algorithm.
2	'learning_rate': [1e-2, 1]	Shrinks the contribution of each tree to *learning_rate*. There is a trade-off between *learning_rate* and *n_estimators* (which has been left to run with default attributes (*n_estimatorsint=100*)).

3	'min_samples_leaf': [1,200]	Reviews the minimum number of samples required to be at each leaf node. A split point at any depth is only considered if it leaves at least the defined *min_samples_leaf* training samples in each of the left and right branches.
4	'max_depth': [1,10]	Defines the maximum depth of the individual regression estimators. The maximum depth also limits the number of nodes in the tree.
5	'max_leaf_nodes': [None]	Helps to grow trees with unlimited number of leaf nodes.
6	'validation_fraction': [0.01, 0.31, 0.01]	Defines the proportion of training data to set aside as the validation set for early stopping. The value is kept between 0 and 1, and only used if *n_iter_no_change* is set to an integer.
7	'n_iter_no_change': [1,20]	Decides if early stopping will be used to terminate training when the validation score is not improving. The number to which it is set, determines a set aside *the validation_fraction* size of the training data as validation and terminate the training when validation score is not improving in all of the previous *n_iter_no_change* numbers of iterations, while keeping the split as stratified.
8	'tol': [1e-7]	Gives tolerance for the early stopping of the learning process when the loss is not improved by at least the given *tol* for *n_iter_no_change* iterations (if set to a number), the training stops.

Table S6: XGBClassifier for XGB and its optimization hyperparameters.

#	**Hyperparameter attributes**	**Description**
1	'booster': ['gbtree']	This parameter specifies that the booster to be used should be tree-based models.
2	'objective': ['binary:logistic']	This parameter refers to logistic regression for binary classification in output probability.
3	'verbosity': [0]	This parameter describes the degree of verbosity of printing messages, it is set to 0 for silent.
4	'reg_lambda': [1e-8, 1.0]	This describes the L2 regularization (ridge regression) term on weights for determining how conservative the model should be.
5	'alpha': [1e-8, 1.0]	This describes the L1 regularization (lasso regression) term on weights for determining how conservative the model should be.

6	'eta': [1e-8, 1.0]	Step size shrinkage used in update to prevent overfitting. After each boosting step, weights of new features can directly be obtained, and *eta* shrinks the feature weights to make the boosting process more conservative.
7	'gamma': [1e-8, 1.0]	Minimum loss reduction required to make a further partition on a leaf node of the tree.
8	'max_depth': [1, 30]	This parameter determine the maximum tree depth for base learners to manage the complexity of the model and avoid overfitting and also manage memory consumption by XGBoost classifier while training a deep tree.
9	'grow_policy': ['depthwise', 'lossguide']	This parameter determines the tree growing policy, or how new nodes are added to the tree. The *depthwise* choice splits at nodes closest to the root, while *lossguide* splits at nodes with highest loss change.
10	'n_estimators': [10,1000]	This determines the number of boosting rounds.
11	'min_samples_split' : [2, 50]	Describes the minimum number of samples required to split an internal node.
12	'min_samples_leaf': [1, 50]	Reviews the minimum number of samples required to be at each leaf node. A split point at any depth is only considered if it leaves at least the defined *min_samples_leaf* training samples in each of the left and right branches.
13	'subsample': [0.5, 1.0]	This determines the subsample ratio of the training instances. Setting it to 0.5 means that XGBoost would randomly sample half of the training data prior to growing trees. and this will prevent overfitting so that subsampling occurs once in every boosting iteration.
14	'min_child_weight': [0.1, 10]	This is the minimum sum of instance weight (hessian) needed in a child tree. If the tree partition step results in a leaf node with the sum of instance weight less than *min_child_weight*, then the building process will give up further partitioning. Higher value of this parameter leads to a more conservative model.
15	'colsample_bytree': [0.1, 1.0]	This is the subsample ratio of columns when constructing each tree so that subsampling occurs once for every tree constructed.
