# Supplementary figures and images for: Predicting Mortality in Hospitalized COVID-19 Patients in Zambia: An Application of Machine Learning
Source: Glob Health Epidemiol Genom. 2023 May 22;2023:8921220. doi: 10.1155/2023/8921220 (PMC10228226; doi:10.1155/2023/8921220)

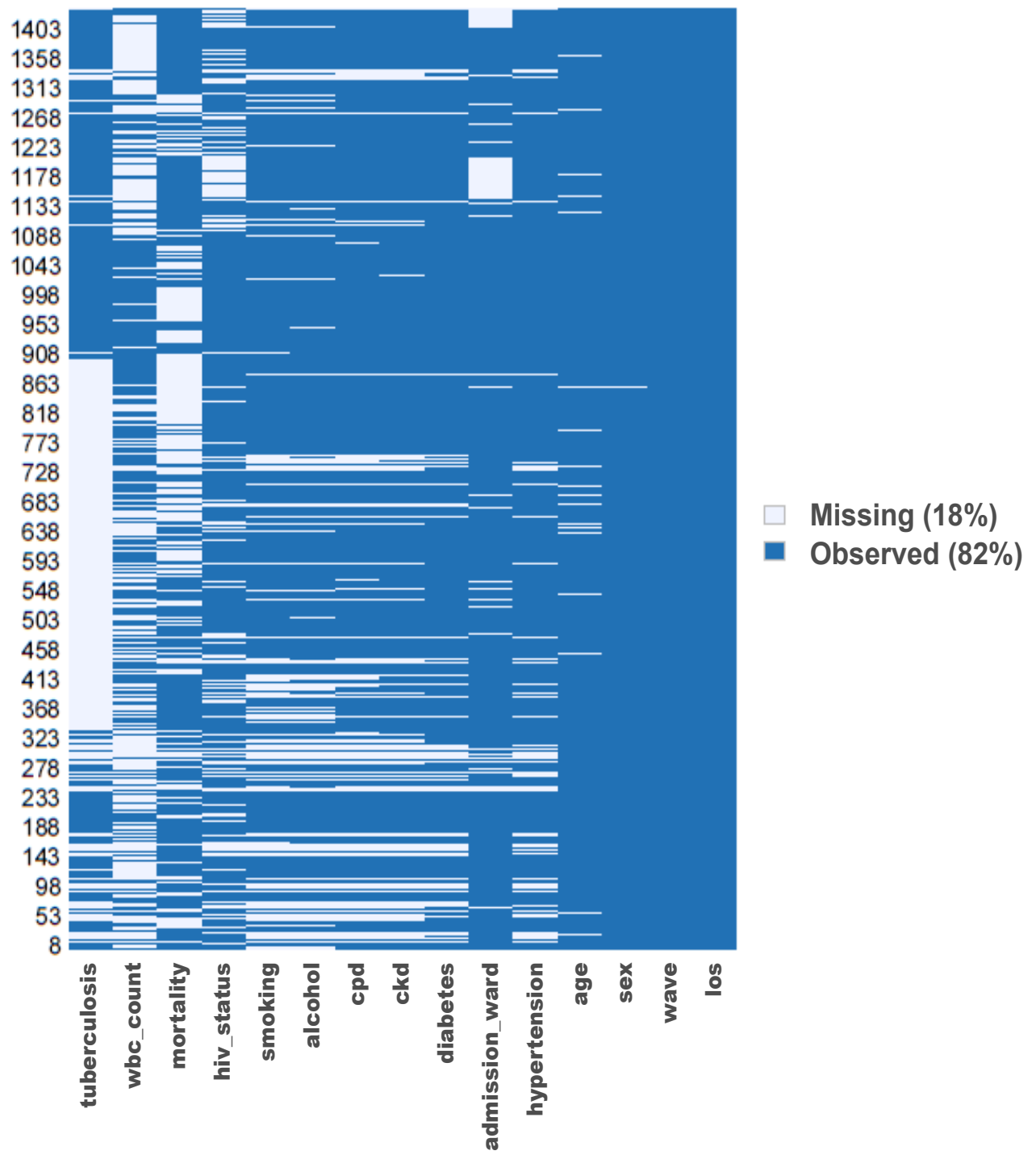

Supplement: Supplementary Materials — Supplementary Material 1: supplementary material 1 (PDF file (.pdf), 46.6 kB-“Figure S1-Dataset missingness map.pdf”) is a figure (Figure S1), and it shows the level of missingness shown by a missingness map, which was produced before multiple imputations by chained equations (MICE) were performed. Supplementary material 2: supplementary material 2 (PDF file (.pdf), 176.0 kB-“ML Models Optimization Hyperparameters.pdf”) contains guidelines in table form about how the hyperparameter tunings and their attributes were set in model development to optimize for best performance for each of the seven ML models validated. Table S1 shows the hyperparameters for the decision tree classifier for the DT model. Table S2 contains hyperparameters for the random forest classifier for the RF model. Table S3 contains hyperparameters for the SVC (support vector classifier) for the SVM model. Table S4 contains hyperparameters for the logistic regression classifier for the LR model. Table S5 contains hyperparameters for the gradient boosting classifier for the GB model. Finally, Table S6 contains hyperparameters for the XGB classifier for the XGB model. [file 8921220.f1.zip › Figure S1.pdf]
